# Selection for the Public Service

**Published:** 1905-04-08

**Authors:** 


### The Hospital.

A Journal of

The Medical Sciences and Hospital Administration.

Vol. XXXVIII.—No. 967. SATURDAY, APRIL 8, 1905.

### Selection for the Public Service.

The recently issued reports of the committees engaged in the selection of boys for admission as Naval Cadets appear to show not only that the system now adopted is a thoroughly sound one, but also that it may be regarded as a first step in the practical application of physiology to the requirements of the public services, and may therefore be hailed with especial satisfaction by the members of the medical profession. The selection of candidates for the services, whether naval, military, or civil, has hitherto been governed either by patronage or by competitive examination in literary or scholastic subjects. In favour of the former it may unquestionably be urged that the friends of a man in authority are just as likely to be capable and trustworthy as the friends of other people; and that the country has been indebted to patronage for the officers who have carried it safely through the greatest struggles in which it has been engaged. Notwithstanding this, the method is hopelessly out of favour with the present generation, and no return to it is even conceivable. It would practically exclude a large number of possibly deserving persons, and would thus unduly limit the range of choice of those in authcrity. The first substitute for it that was suggested or put in practice was that of competitive scholastic examination; an expedient of the Gradgrind school which everyone capable of judging must from the first have known to be foredoomed to failure, and which was probably only suggested by Ministers and accepted by Parliament as a sop to the so-called democratic tendencies of the day. The competitive examination delivered over the services to the hands of the crammer, and afforded no sort of test of the qualities calculated to fit the candidates for the duties actually to be imposed upon them. The son of a very gallant and distinguished officer was rejected for the army, and his father, after careful scrutiny of the examination papers, discovered that the boy's failure was due to his want of knowledge of the career of Anselm, an Archbishop of Canterbury in the eleventh century, whose achievements are more frequently mentioned in theological than in military circles. The father went straight to the head of his profession. "Can you tell me, Sir, who Anselm was?" The great personage bestowed a benediction, and admitted his incapacity. "Then the knowledge is not essential to a soldier?" The answer may be conceived, and, in due course, the case of the son was reconsidered. Not every rejected candidate, however, possessed a friend capable of taking his case to headquarters in so effective a manner, and old non-commissioned officers were for years accustomed to describe the young men who came to their regiments as "heart-breaking." The war in South Africa found some of them in positions which they had presumably attained by seniority, and thus afforded a crucial test of the merits of the system under which they had originally found entrance into the service.

The Admiralty and, for the matter of that, the country, is to be congratulated on having at last discovered a more excellent way. Candidature for naval cadetships is open to all boys, but the candidates are examined conversationally instead of competitively, and the examinations are conducted by committees composed of naval officers and schoolmasters, who found their judgments upon aspect, bearing, and manifest intelligence, instead of upon written answers to stereotyped questions. Gentlemen and men of the world would put a boy at his ease in five minutes, and in very few more would be able to form an opinion of his general fitness for the service—of the presence or absence, that is to say, of the substratum of essential qualities which it requires. If accepted by the committee, a boy has still to undergo a medical examination, and to show ordinary proficiency in the school work to be expected at his age. This method has now been two years in operation, and its success is admitted in the most unqualified terms by all whose opportunities have enabled them to judge. As we have already suggested, it is thoroughly physiological in its character, and manifestly deserves to be regarded as a practical application of science to the wants of daily life. Every physiologist would declare the competitive examination test to be thoroughly unsound and untrustworthy, and certain to prove but a broken reed in the hands of all who leant upon it.

We would, however, suggest that the new system, excellent as it seems to be, might be brought more into harmony with scientific knowledge by the application to it of Mr. Francis Galton's doctrines of heredity and of "eugenics." Much of the best work in English naval and military history has been accomplished by the members of what may be called "service families"—that is to say, by men deriving special aptitudes from descent. It is only reasonable that the possessors of such aptitudes should be chosen whenever possible, and all that would be necessary for this purpose would be to give the sons of naval officers preferential admission before the examining committee, and also a preference over other candidates in cases of equality with a limited number of vacancies. Such a course would not only be likely to secure the best officers, but it would also bring to the service concerned the almost incalculable advantages of tradition. No one who knows the soldier can entertain any doubt that the "Die-hards" derived additional strength at Inkerman from the knowledge that they were led by the son of the man under whose command the regiment won its glorious title at Albuera; nor can anyone doubt that English sailors would rejoice in being taken into action by the kinsman of the naval heroes of whom their fathers had spoken to them.

